# The speed-curvature power law in tongue movements of repetitive speech

**DOI:** 10.1371/journal.pone.0213851

**Published:** 2019-03-18

**Authors:** Stephan R. Kuberski, Adamantios I. Gafos

**Affiliations:** Department of Linguistics and Research Focus Cognitive Sciences, University of Potsdam, Potsdam, Brandenburg, Germany; Universite Sorbonne Nouvelle Paris 3, FRANCE

## Abstract

The speed-curvature power law is a celebrated law of motor control expressing a relation between the kinematic property of speed and the geometric property of curvature. We aimed to assess whether speech movements obey this law just as movements from other domains do. We describe a metronome-driven speech elicitation paradigm designed to cover a wide range of speeds. We recorded via electromagnetic articulometry speech movements in sequences of the form /CV…/ from nine speakers (five German, four English) speaking at eight distinct rates. First, we demonstrate that the paradigm of metronome-driven manipulations results in speech movement data consistent with earlier reports on the kinematics of speech production. Second, analysis of our data in their full three-dimensions and using advanced numerical differentiation methods offers stronger evidence for the law than that reported in previous studies devoted to its assessment. Finally, we demonstrate the presence of a clear rate dependency of the power law’s parameters. The robustness of the speed-curvature relation in our datasets lends further support to the hypothesis that the power law is a general feature of human movement. We place our results in the context of other work in movement control and consider implications for models of speech production.

## Introduction

Speech is perhaps “the most highly developed motor skill possessed by all of us” [1]. The continuous deformations of the vocal tract structuring the sound of speech involve the precise positioning of a number of articulatory organs as they form and release constrictions in a limited space inside the body. Speech has evolved to harness this complex activity for the purposes of communication. A remarkable fact about the robustness of the resulting system is that what a linguist considers to be the same utterance can be conveyed by different individuals under wildly different conditions. For example, age, gender, size, loudness, and speed all contribute to the formation of the speech sounds which are then recovered as an instance of e.g., [ta] or [ka]. Given the remarkable variability of conditions under which speech goals are achieved, the identification of invariances (at best, laws) in kinematic characteristics of speech movements has been seen as an imperative [2, 3]. The identification of such invariances offers crucial criteria for model evaluation. Any proposed model of speech production must conform to these. Furthermore, despite the relatively early influence of concepts and in some cases models from general motor control had on models of speech production [4–7], our understanding of the extent to which speech movements conform to well-known laws from other areas of human movement is at its infancy [8–10].

Results from speech hold potential for implications in the other direction, that is, from speech to theories of biological action in general. A recurring debate in motor control concerns the neurobiological bases of any given law. Opposing perspectives range from laws as consequences of direct cortical computations [11], to laws as consequences of coupling among neural and physical (limb) dynamics [12] and or viscoelastic muscle properties [13], and even sometimes to laws as mere computational artifacts [14]. The human tongue is unlike the arm or the finger in that it is not supported by a skeletal system. Biomechanically, tongues, trunks of elephants and tentacles of cephalopods [15] are muscular hydrostats, that is, structures whose volumes remain constant across transformations in shape. Control of the hydrostat’s shape is crucial to speech. Making a [ta] or a [ka] involves bending the muscular hydrostat by simultaneously contracting longitudinal and antagonistic circular, transverse or radial muscles. Overall, then, the control of limb versus tongue seems to be qualitatively different [16]. Knowledge of whether principles that organize and constrain the functioning of other effectors also apply to the tongue is thus likely to provide more clues on the bases of such principles. In the event that a law generalizes across tongues and limbs, this indicates that any proposed bases for that law must be general enough to encompass invariance with respect to the identity of the effectors and thus presumably downstream specifics of their mode of operation.

The power law relation between speed of movement and curvature of effector trajectory is a celebrated law of human motor control. Consider drawing an ellipsis with a stylus or a finger. According to this law, as the stylus traces the trajectory segments of the ellipsis where curvature increases, movement speed systematically slows down. As the curvature of the traced segments decreases, speed increases. The law is called a power law because one quantity, here speed, varies as an exponential function, that is, a power of another quantity, here curvature. We aimed to assess whether speech movements obey this law. Explicit control of curvature in (speech) articulatory motion is not as feasible as in, for example, drawing tasks where the experimenter can prescribe at least the gross geometric features of the required movements such as in drawing a straight line or an ellipsis or a more complex, say, figure-eight-like pattern. In our assessment, thus, we capitalized on control of speed, the other essential quantity of the law in question. Using a metronome-driven speech elicitation paradigm, we aimed to elicit as great a range of movement speeds as possible. Specifically, building on the paradigm of repetitive speech [10, 17, 18], we recorded via three-dimensional electromagnetic articulometry (EMA), speech movements in sequences of the form /CV…/ from nine adult speakers (five were native speakers of German and four were native of English) speaking at eight distinct rates ranging from extremely slow (30 beats per minute, bpm) to extremely fast (570 bpm). In the resulting dataset, we sought evidence of the sort that has provided support for the power law in other areas of motor control.

We show, first, that despite the extensive rate variation implemented in our experimental paradigm, the resulting data conforms to well known kinematic properties of speech. In turning to evaluate the speed-curvature power law, we take into account all three-dimensions of our registered data. Prior work on the law in non-speech domains and most work in the speech domain has so far either restricted movements in two dimensions via task-constraints, as in drawing on a plane where the relevant data are only those generated by contact between a stylus and a two-dimensional surface, or reduced what are naturally three-dimensional movements to two dimensions via method constraints by using previous generation two-dimensional electromagnetic articulometry systems. Our evaluation of the law is based on three-dimensions. Specifically, we demonstrate that using the full dimensionality of the movements and advanced numerical differentiation methods (as crucially required for the accurate estimation of curvature in 3D) substantially improves the significance of the findings in comparison to earlier reports while obviating the exclusion of data subsets with certain curvature values (as done in some of the earlier reports on the law). Furthermore, we demonstrate the presence of a clear speech rate dependency of the power law’s parameters. The robustness of the law in our datasets lends further support to the hypothesis that the power law is a general feature of human movement. The modulation of the law’s parameters by rate likely reflects geometric properties of effector trajectory shape. Such modulation is in agreement with other results from non-speech domains which have uncovered links between the geometry of a movement and the specific parameterization of the law as well as, under some proposals, between the geometry of a movement and the underlying control regime effecting that movement.

### Background

Theoretically, the goal of moving an effector from one point in space to another can be achieved in an infinite number of ways. However, in biological action and human movement more specifically [19], the motor system does not make use of the entire set of available paths in such point to point movements. Rather, the system seems to give preference to certain paths over others by exploiting invariances which effectively reduce the number of degrees of freedom. One such invariance is expressed by a relation between movement speed and trajectory curvature. When an effector traces a trajectory, the speed of the effector’s movement decreases at the more curved parts of a trajectory and increases as the trajectory becomes straighter. This speed-curvature relation derives from observations on hand drawing and writing movements [20]. In extension of this work to other settings, evidence for the law was reported also for movements of the oculomotor system evidenced in eye-tracking experiments [21] as well as for movements of the lower limb locomotion system [22]; for a recent encompassing review see [23]. There is also evidence that the loss of function implicated in limb apraxia results in degradation of the relation between speed and curvature [24–27]. The law has been shown to hold not only during performance of various actions but also in perceiving those actions. When visual stimuli are designed to violate the law, perception of the portrayed motions is faulty or distorted in a direction that conforms to the law [19]. Furthermore, observing stimuli obeying the law elicits stronger activations in brain areas linked to visual processing, action production and action perception than stimuli violating the law [28].

As first proposed by Lacquaniti et al. [29], the proposed relation between instantaneous speed of movement *v* and trajectory curvature *κ* reads

$$\begin{array}{c} v=k\kappa^{-\beta } \end{array}$$ (1)

with empirically determined exponent *β* and so-called velocity gain factor *k*. Thus, movement speed is a power function of trajectory curvature. Past work in non-speech domains suggests that, under certain assumptions about the class of shapes traced by the studied effector, the exponent *β* is generally close to a value of one third (hence, the law is often referred to as the one-third power law) and that *k* is constant within identifiable long segments of a trajectory but discontinuously changes at inter-segmental transitions [22, 30].

A few attempts have been made in assessing the presence of the speed-curvature power law in the domain of speech. Tasko & Westbury [31] analyzed two-dimensional articulatory data from nine male and nine female American English speakers registered using an x-ray microbeam system. The participants were asked to read sentences at a self-selected speech rate. Movement speed and trajectory curvature were found to be related by a power law with an exponent near to the value of one third, with some indication of different articulators exhibiting variations in the precise expression of the law. Perrier & Fuchs [32] investigated data registered using two-dimensional midsagittal electromagnetic articulometry from six speakers of different languages (French, German and Mandarin Chinese). The study’s reading task contained several vowel-vowel and vowel-consonant-vowel combinations. In addition to these data, the authors analyzed data simulated by a biomechanical tongue model at three distinct speech rates (slow, normal and fast). Results showed that the power law offers a fair description of the global speed-curvature relation for all speakers and all languages as well as for the simulated data, however, with a significant inter-speaker variability of the power law’s exponent *β* and velocity gain factor *k*. Neufeld & van Lieshout [33] analyzed three-dimensional articulatory data of six native Canadian English speakers (three females, three males). Prioritizing naturalistic speech, participants were asked to read aloud from a book of short stories. The work reports that the speed-curvature power law holds with an exponent close to one third, though, with differences between articulators similar to the earlier results reported in [31]. Most recently, Tomaschek et al. [34] analyzed articulations of German words of distinct lexical frequencies. In two-dimensional data from 16 speakers (eight female, eight male), the study found support for the presence of the speed-curvature power law. However, it was also reported that for lower values of curvature the effect of curvature on speed levels off substantially. Hints of weakening of the law for low curvature values are also found in [31]. Thus, both latter works excluded extremal values of curvature prior to the regression analyses used in assessing the law in their speech data.

In the background of this prior work, the key characteristic of our present study is the extensive rate manipulation using metronome rates from extremely slow (30 bpm) to extremely fast (570 bpm). This allowed us to assess the speed-curvature law on the basis of data from a (substantially) wider range of speed conditions than in any prior work. It should be evident that because the law refers to speed, a thorough evaluation of its applicability in speech should aim to vary this parameter to the extent possible.

## Methods

Five native speakers of German (three females, two males) and four native speakers of English (three females, one male) participated in the experiment. In the following, aliases G1–G5 refer to the German speakers of our study. Aliases E1–E4 refer to the English speakers. Data from two other English speakers was registered but had to be excluded due to an unnoticed hardware equipment failure while recording. The speakers were between 18 and 35 years old and without any present or past speech or hearing problems. They were recruited at the University of Potsdam and paid for their participation in the experiment. All procedures were performed in compliance with relevant laws and institutional guidelines and were approved by the ethics committee of the University of Potsdam. Written informed consent was obtained from all subjects.

During the experiment, participants were prompted on a computer screen to produce sequences of repeated [ta] or [ka] syllables in time with an audible metronome. The metronome served as an extrinsic index of the intended rate of syllable production. The participants were instructed to articulate their responses accurately and naturally. The rate of the metronome was set to the values of 30, 90, 150, 210, 300, 390, 480 and 570 bpm (corresponding to 0.5, 1.5, 2.5, 3.5, 5.0, 6.5, 8.0 and 9.5 Hz). At the start of each trial, the participant was exposed to the metronome beats and begun articulating the required response syllable at a point of their choice. Intuitively, we can describe this by saying that participants begun uttering repetitive syllables once they had listened to the metronome and internalized the intended rate. Once participants begun uttering, the length of each such trial of repetitive syllable productions consisted in a sequence of approx. 30 syllables. Starting with the slowest rate, a minimum of four trials at each of the above rates were recorded. Once this minimum of four trials was reached, recording proceeded to the next higher rate. The entire procedure was performed in two successive blocks, first for sequences of [ta] and then for sequences of [ka].

Articulatory data as well as acoustic data were registered from all participants. All recordings took place in our sound-attenuated booth using a Carstens AG501 3D Electromagnetic Articulograph for articulatory, and a YOGA Shotgun microphone EM-9600 attached to a TASCAM US-2x2 Audio interface for acoustic data registration. Three-dimensional electromagnetic articulography (EMA) allowed measurement of kinematic displacement data of selected articulators at a high precision. Along with some other auxiliary reference locations (upper and lower incisors, nose bridge, left and right mastoid processes), we tracked the positions of sensors attached to the tongue tip and tongue back articulators, the major effectors involved in the production of [ta] and [ka] respectively. All data and source code files used to produce the results presented below are fully available under doi:10.5281/zenodo.2273898.

### Data processing

Three-dimensional displacement data, provided by the AG501 device, was digitized at a sampling rate of 1250 Hz. In order to reduce storage and memory footprint as well as to improve further data processing performance, the sampling rate of all signals was decreased to a value of 83.33 Hz by means of a double-stage decimation procedure. This decimation procedure included two successive 30th order equi-ripple FIR lowpass filters (with an effective cutoff frequency of 41.67 Hz) eliminating most high frequency noise. Based on the downsampled, noise-reduced signals, spatial transformations of head movement correction and occlusal reference frame alignment were determined and applied by means of the method proposed by Horn [35]. Finally, a zero-delay Butterworth lowpass filter of fourth order with cutoff frequency of 25 Hz was utilized to eliminate any further noise potentially present (see [36]).

All signals were represented analytically by means of a quintic spline approximation. Spline approximation was carried out individually for each dimension at equidistant knots given by the constant sampling rate of 83.33 Hz. Quintic polynomials were determined by a least squares regression of data points and continuity constraints of derivatives up to the third order (see e.g., [37, 38] for a general description of regression splines). This method allowed for the representation of an articulator’s essential kinematic properties (displacement, velocity and acceleration) at the same time by a single analytic spline object. Numerical differentiation was carried out using nine-point finite differences with eighth order of accuracy (stencil coefficients were determined using the Python package [39]).

**Fig 1:**
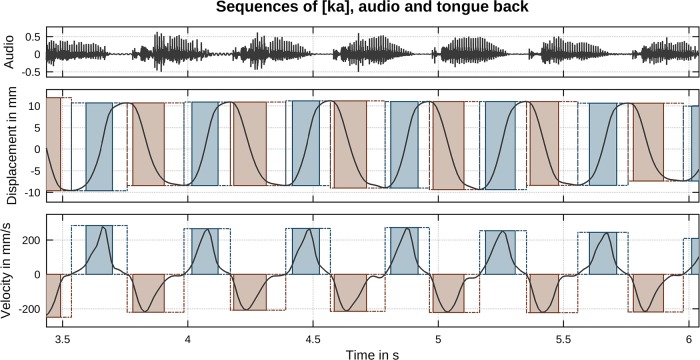
Section of a [ka] sequence at 150 bpm. (Top) Acoustic recording. (Middle) Principal component (PCA) of tongue back displacement. (Bottom) First derivative (velocity) of PCA. Segmented movements are indicated by shaded boxes (closing movements: blue, opening movements: brown). Dashed line rectangles correspond to movement delimiters (zero velocity) and solid line rectangles to movement onsets and offsets determined by 20% peak velocity crossings.

The continuous three-dimensional motion of the tongue back and tongue tip articulators was segmented into separate, successive closing and opening movements. The basis for this segmentation was the first derivative (velocity) of the displacement’s principal component (by means of a PCA, representing displacement along movement direction). Instants of zero-crossings in the PCA velocity were used as primary movement delimiters (see, e.g., [2]). Movement onset and offset locations were determined as the points where velocity rose above (onset) or fell below (offset) 20% peak velocity. In cases where the value of 20% peak velocity was crossed more than twice in a single (closing or opening) movement (multi-peak velocity profiles), we chose the earliest crossing as the movement onset delimiter and the latest crossing as the offset delimiter. The 20% threshold was chosen in order to avoid potentially poorly defined transitions into or away from a quasi-steady-state phase [40]. Segmentation was carried out fully automatically. A final, manual task was performed by selecting only those movements directly involved in forming or releasing tongue-palate constrictions neglecting any other, non-targeted movements potentially present (movements between syllables which did not contribute to any acoustic output, primarily present at the slowest rate, were neglected). As an example of our data, Fig 1 shows a series of [ka] syllables produced by one of our participants at a metronome rate of 150 bpm. In total, we registered 12 738 movements in the [ta] case (6 284 closures and 6 454 openings) and 12 379 movements in the [ka] case (6 119 closures and 6 260 openings).

## Results

In the context of previous work on the speed-curvature relation, the distinguishing property of our experimental design is the extensive range of rates under which participants spoke their utterances. Our participants spoke syllables at eight distinct rates ranging from extremely slow (30 bpm) to extremely fast (570 bpm). For comparison, in prior work on repetitive speech, Kelso et al. [17] included two rates (speaker-selected conversational and fast), as did Ostry et al. [10] (approx. one syllable and two syllables per second), and Patel et al. [18] used a metronome to suggest a rate to their participants (which was 120 bpm) but there was no metronome during the actual registration of a participant’s utterances. In previous work on the speed-curvature power law, Perrier & Fuchs [32] considered three rates (slow, normal and fast) in target-to-target biomechanically simulated tongue movements. Tomaschek et al. [34] investigated two speech rates (slow and fast) by control of inter-stimulus and stimulus presentation times. In light of our extensive speech rate manipulation, it is thus imperative to first ensure that our so-registered data are in conformity with what is known about speech movements from earlier work.

For any movement, the three basic kinematic properties are those of movement duration *T*, movement amplitude *A* and peak velocity v* [8]. In our data, movement duration was determined as the time between movement onset and offset based on 20% peak velocity delimiters. Movement amplitude was computed as the length of the trajectory the effector moved in three-dimensional space. Peak velocity was determined as the maximum magnitude of tangential velocity

$$\begin{array}{c} v=\sqrt{{\dot{x}}^{2}+{\dot{y}}^{2}+{\dot{z}}^{2}}, \end{array}$$ (2)

where $\dot{x}$, $\dot{y}$ and $\dot{z}$ denote the first derivatives of the horizontal, vertical and lateral components of the effector’s position in space.

**Fig 2:**
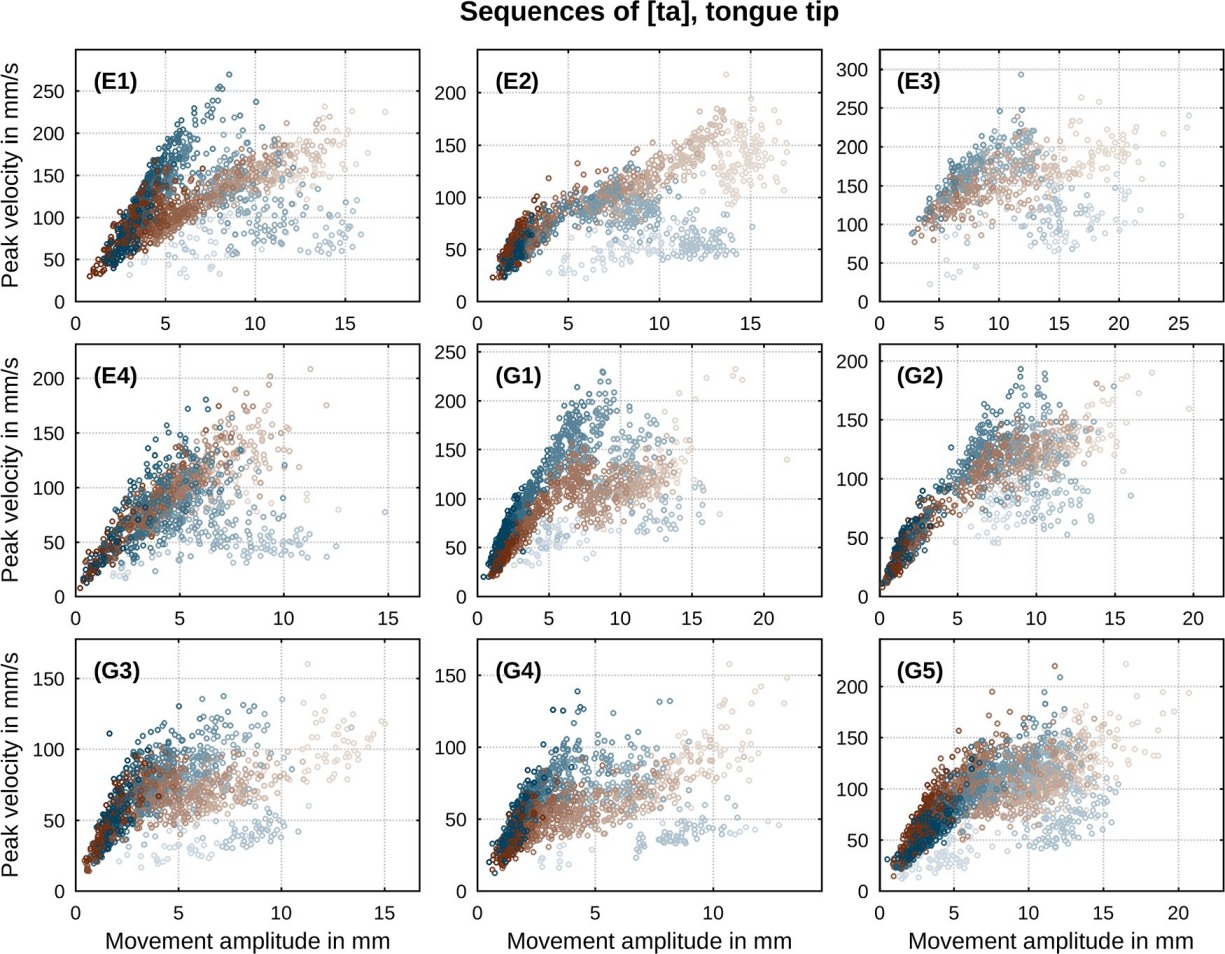
Per-speaker relation between peak velocity and movement amplitude in sequences of [ta]. Direction of movement is indicated by color (closing: blue, opening: brown). Metronome rate is indicated by gradual shade (slowest: bright, fastest: dark).

**Fig 3:**
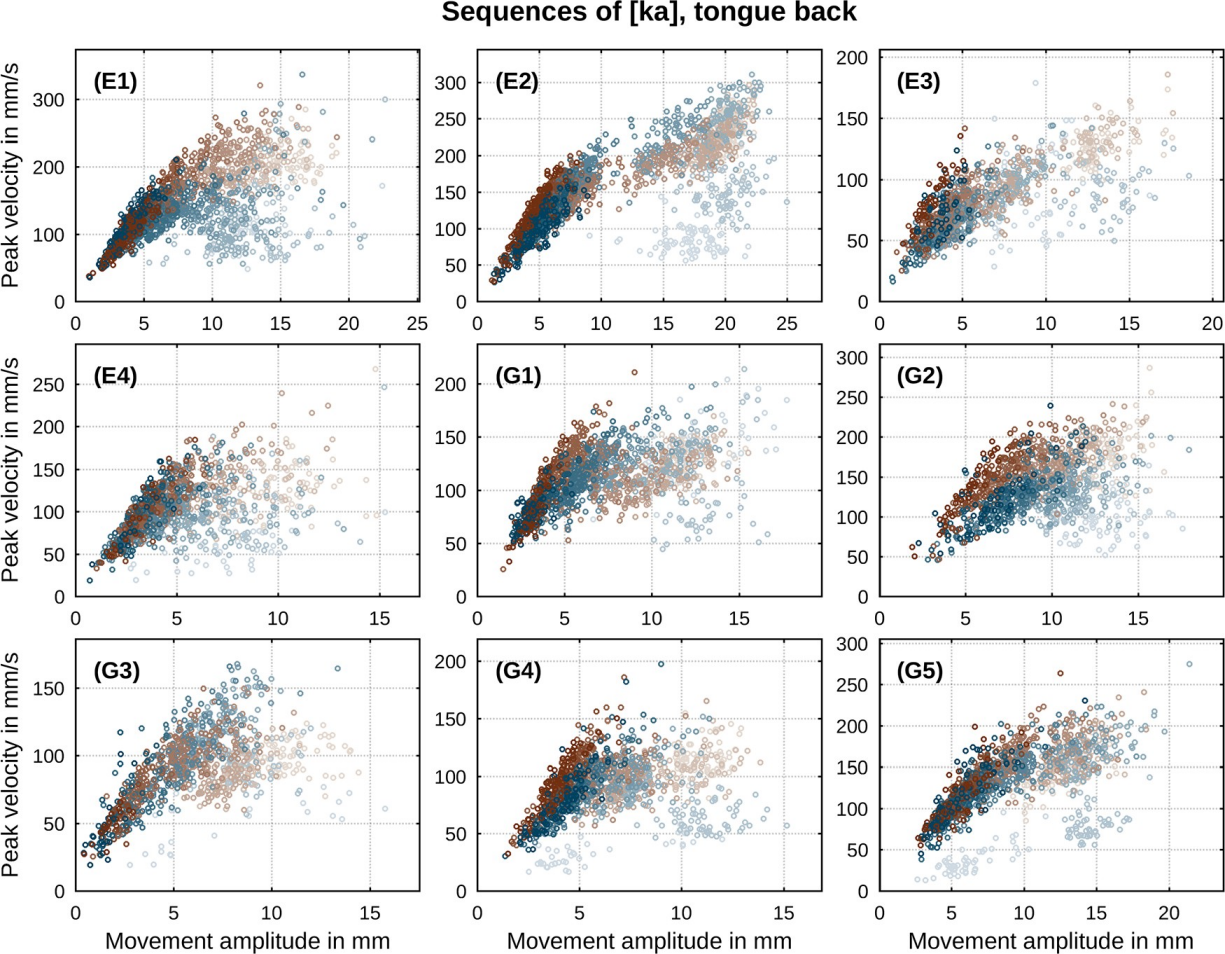
Per-speaker relation between peak velocity and movement amplitude in sequences of [ka]. Direction of movement is indicated by color (closing: blue, opening: brown). Metronome rate is indicated by gradual shade (slowest: bright, fastest: dark).

One empirically well-documented relation between the above kinematic parameters is the correlation between movement’s peak velocity v* and its amplitude *A*. It has been reported for a large variety of consonant-vowel (CV) and vowel-consonant (VC) sequences involving movements of tongue body, tongue tip, lips and jaw [41]. The relationship has been described as an overall linear correlation [42] with *A*-v* slopes steeper for faster than for slower speech rates [43, 44] and decreasing covariation as durational variability increases (ibidem). Evidence for divergence from linearity at larger amplitudes is known from previous data and has been of concern in previous modeling studies (e.g., [45]). Figs 2 and 3 show per speaker *A*-v* scatter plots of our data in sequences of [ta] and [ka]. For both sequences there is an overall correlation between peak velocity and movement amplitude with divergence from linearity at larger amplitudes. In addition, we observe substantially steeper correlation slopes of *A* and v* for faster speech rates (indicated by darker shades) than for slower rates (indicated by brighter shades). Thus, the characteristics of the *A*-v* relation in our data are in broad conformity with earlier reports across the entire range of induced speech rates in our paradigm.

**Fig 4:**
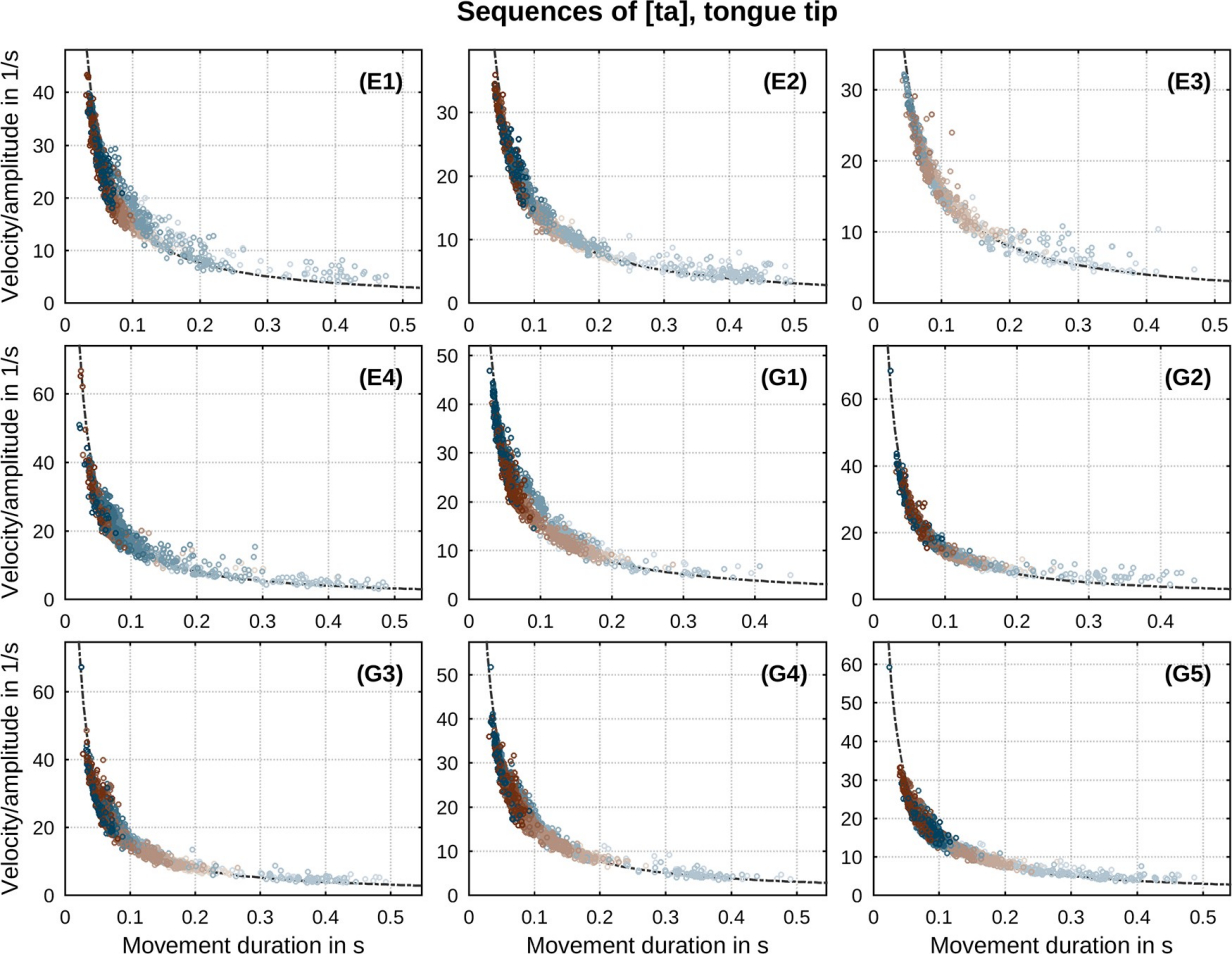
Per-speaker relation between ratio of peak velocity to movement amplitude and duration in sequences of [ta]. Direction of movement is indicated by color (closing: blue, opening: brown). Metronome rate is indicated by gradual shade (slowest: bright, fastest: dark). Regression lines according to v*/*A* ∝ 1/*T* are drawn as black curves.

**Fig 5:**
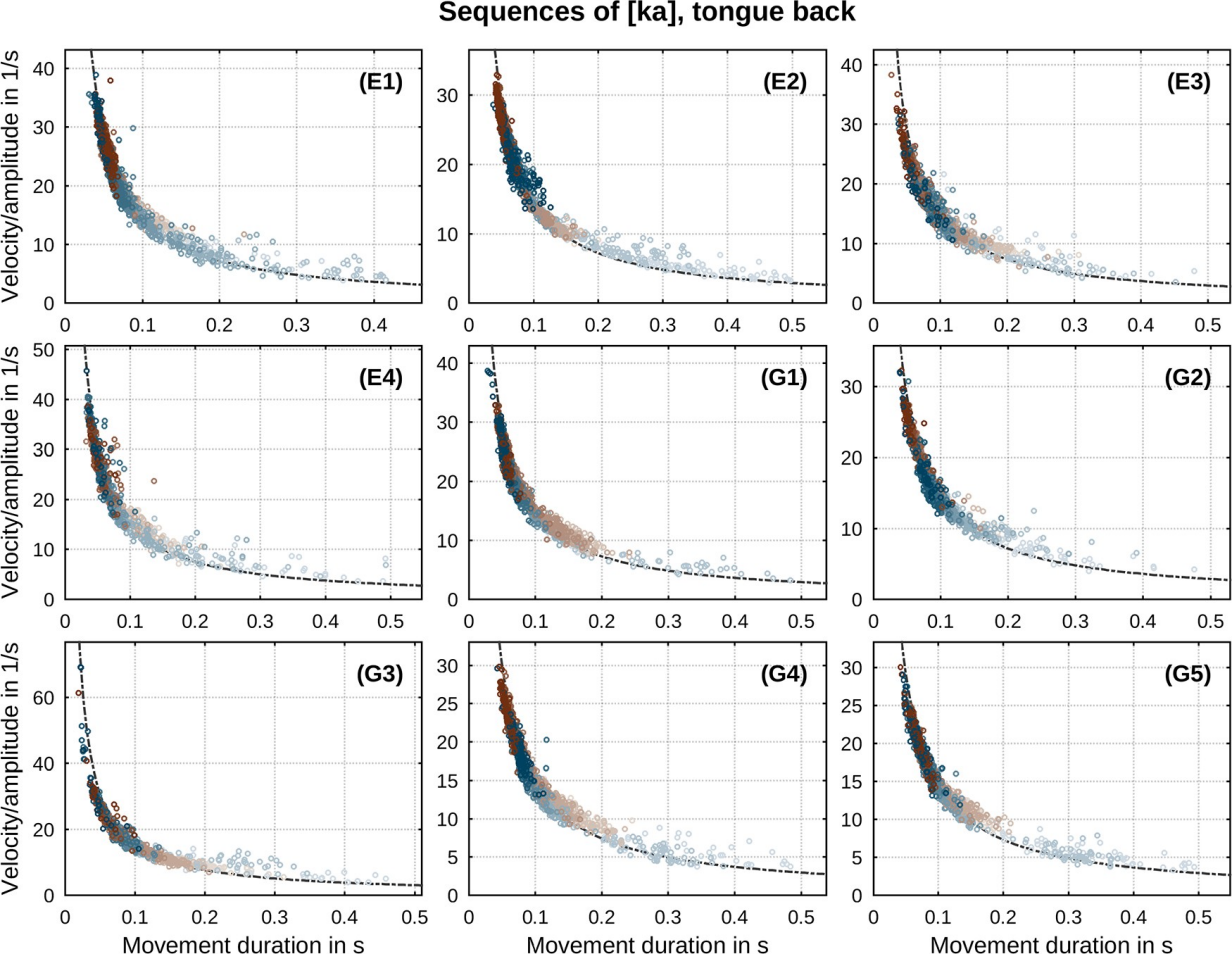
Per-speaker relation between ratio of peak velocity to movement amplitude and duration in sequences of [ka]. Direction of movement is indicated by color (closing: blue, opening: brown). Metronome rate is indicated by gradual shade (slowest: bright, fastest: dark). Regression lines according to v*/*A* ∝ 1/*T* are drawn as black curves.

Another prototypical feature of speech movements is the presence of a relation between all three kinematic parameters considered. It has been commonly reported that the ratio of peak velocity to amplitude v*/*A* varies inversely with movement duration *T* (e.g., [2]) across manipulations of stress, vowel and consonant identity [42, 46]. The relations found in our data are shown in Figs 4 and 5 for both sequences of [ta] and [ka]. Regressions of the inverse correlation v*/*A* ∝ 1/*T* are drawn in black color. Values of correlation slopes separated by movement direction are listed in Table 1 (for direct comparison with the constant factor *c* in Eq (4) these values are divided by *π*). In conformity with past observations, our data exhibit a clear inverse correlation between the ratio of peak velocity to movement amplitude and movement duration. Deviations from the overall relationship at longer movement durations (slower metronome rates, indicated by brighter shades) have been previously reported by Ostry & Munhall [42] and related to changes in articulator stiffness.

**Table 1:** Per-speaker regression slopes (in terms of *π*) of the inverse correlation between the ratio of peak velocity to amplitude and movement duration. Values are separated by sequence and movement direction.

	E1	E2	E3	E4	G1	G2	G3	G4	G5
[ta] closing	0.515	0.515	0.516	0.538	0.507	0.499	0.508	0.487	0.484
[ta] opening	0.454	0.474	0.505	0.479	0.477	0.470	0.489	0.485	0.471
[ka] closing	0.461	0.471	0.467	0.482	0.451	0.458	0.493	0.462	0.462
[ka] opening	0.460	0.451	0.471	0.475	0.476	0.455	0.482	0.482	0.471

Both empirical relations discussed here are in good agreement with assumptions about the control system governing speech gestures. A long line of work has proceeded on the hypothesis that the units of action underlying the flow of speech movements are controlled by an organization similar to a mass-spring system [4–6]. The standard model of the gesture is a special case of the damped linear oscillator with critical damping or more specifically

$$\begin{array}{c} \ddot{x}=-kx-b\dot{x},\;b=2\zeta \sqrt{k},\;\zeta =1, \end{array}$$ (3)

where stiffness *k* (not to be confused with the velocity gain factor in Eq (1) earlier) acts as the model control parameter. Critical damping is realized by a damping ratio of *ζ* = 1 which also fixes the damping constant *b* of the model. Analytical solutions of the dynamical system in (3) can be derived by means of methods from the theory of ordinary differential equations. Analysis of these solutions reveals the following relations between the kinematic properties of peak velocity v*, movement amplitude *A* and movement duration *T*

$$\begin{array}{c} v^{*}=c\sqrt{k}A\;\text{and}\;\frac{v^{*}}{A}=\frac{c\pi }{T}. \end{array}$$ (4)

These are a linear correlation between peak velocity v* and movement amplitude *A* (equation on the left) and an inverse relation between the ratio of peak velocity to amplitude and movement duration *T* (equation on the right) sharing the same constant factor *c* (if *π* is factored out as in the right equation).

It can be analytically proven that the factor *c* is upper-bounded by the value of one half [46]. Table 1 lists the per-speaker determined factors *c* of our data. It is evident that there is general agreement between empirical observation and theoretical prediction. In other words, *c* ≲ 1/2 for almost every observation. However, we also find differences between the two directions of movement (closing and opening movements). Factors *c* of closing movements generally attain values larger than those of opening movements, revealing a moderate asymmetry between the two types of direction. As we will show below, a parallel asymmetry exists with respect to properties of the conjectured power law.

### Speed-curvature power law

We now turn to the assessment of the speed-curvature power law. In examining the relation between speed and curvature in our datasets, the full three-dimensional trajectories of the articulators were considered. Let us begin by reviewing the definitions of the two essential variables that play out in the speed-curvature power law. The instantaneous speed *v* of an effector moving in three-dimensional space is given by its tangential velocity expressed in Eq (2). According to the theory of the differential geometry of curves (e.g., [47]), the time-dependent curvature *κ* of a three-dimensional trajectory is given by

$$\begin{array}{c} \kappa =\sqrt{\frac{{\left(\dot{y}\ddot{z}-\ddot{y}\dot{z}\right)}^{2}+{\left(\dot{z}\ddot{x}-\ddot{z}\dot{x}\right)}^{2}+{\left(\dot{x}\ddot{y}-\ddot{x}\dot{y}\right)}^{2}}{{\left({\dot{x}}^{2}+{\dot{y}}^{2}+{\dot{z}}^{2}\right)}^{3}}} \end{array}$$ (5)

with $\ddot{x}$, $\ddot{y}$ and $\ddot{z}$ being the second derivatives (acceleration) of its displacement components. The curvature of a trajectory is a measure of how much a current segment of the trajectory deviates from a straight line. Large values of curvature indicate strongly curved segments whereas values close to zero imply close to straight segments. For the present analysis, we computed speed and curvature values by subsampling each movement’s trajectory at five equidistant locations based on zero velocity onset and offset delimiters. It is usual practice that movement onset and offset delimiters are adjusted to coincide with the two instants of 20% peak velocity (or some such similar percentage). This adjustment is effectively a length reduction of movements yielding gaps in the sequence of successive movements (with gaps typically not included in any analysis). These gaps, the transitions between movements of opposite direction, are generally regions of high curvature. Hence, it is not advisable to suppress these gaps (by adjusting the delimiters) in the assessment of the speed-curvature power law. In total we computed approx. 125 000 pairs of speed and curvature values.

The majority of works on the speed-curvature power law in speech have so far considered only two-dimensional, mid-sagittal data. This is due to either method limitations (e.g., x-ray microbeam provided only two-dimensional displacements) or intentional projection of three-dimensional data onto the mid-sagittal plane. The same applies to many works from the field of general motor control which studied two-dimensional movements by physically restricting a participant’s action to a plane. However, the speed-curvature power law itself is not restricted to planar movements [33, 48, 49]. In our data, across all speakers and rates, angular dispersions away from the mid-sagittal plane are in the range of ±5–10° away from that plane and movement displacements perpendicular to the mid-sagittal plane are in the range of 5–10% of displacements along that plane. In sum, lateral components are relatively insignificant. Nevertheless, even insignificant movement in the lateral dimension affects the computation of curvature in 3D as given by Eq (5) specifically so that its value is different from that in 2D. Hence, exactness calls for usage of the proper three-dimensional expressions of speed and curvature as given by Eqs (2) and (5). Furthermore, computation of curvature (either two-dimensional or three-dimensional) requires precise information about the second derivative of each component’s displacement. High-order numerical differentiation is highly sensitive to noise (the higher the order of the derivative, the more so). Hence, a robust and numerically stable algorithm is required. In addition, expression (5) contains multiple products of first and second derivatives in the fraction’s numerator. The sum of these products is related to the cross product $\dot{r}\times \ddot{r}$ of the vectors $\dot{r}=\left(\dot{x},\dot{y},\dot{z}\right)$ and $\ddot{r}=\left(\ddot{x},\ddot{y},\ddot{z}\right)$. Here too, then, to maintain computational accuracy it is crucial that the numerical derivative of $\dot{r}$ precisely approximates $\ddot{r}$. With these strictures in mind, in our data representation, we utilized nine-point stencil differentiators for high precision numerical differentiation as well as single uniform quintic spline objects for the consistent approximations of *r*, $\dot{r}$ and $\ddot{r}$ at the same time.

As seen in Eq (1), the speed-curvature law states that the speed of movement is a power function of curvature of the related trajectory. An equivalent formulation, in which Eq (1) has been transformed by taking the logarithm of both sides, is given by

$$\begin{array}{c} \log v=\log k-\beta \log\kappa . \end{array}$$ (6)

This is a straight line in the log *κ*-log *v* plane whose slope corresponds to the negative exponent −*β* and whose intercept corresponds to log *k*, with *β* and *k* the two empirically-determined constants of the conjectured power law.

**Fig 6:**
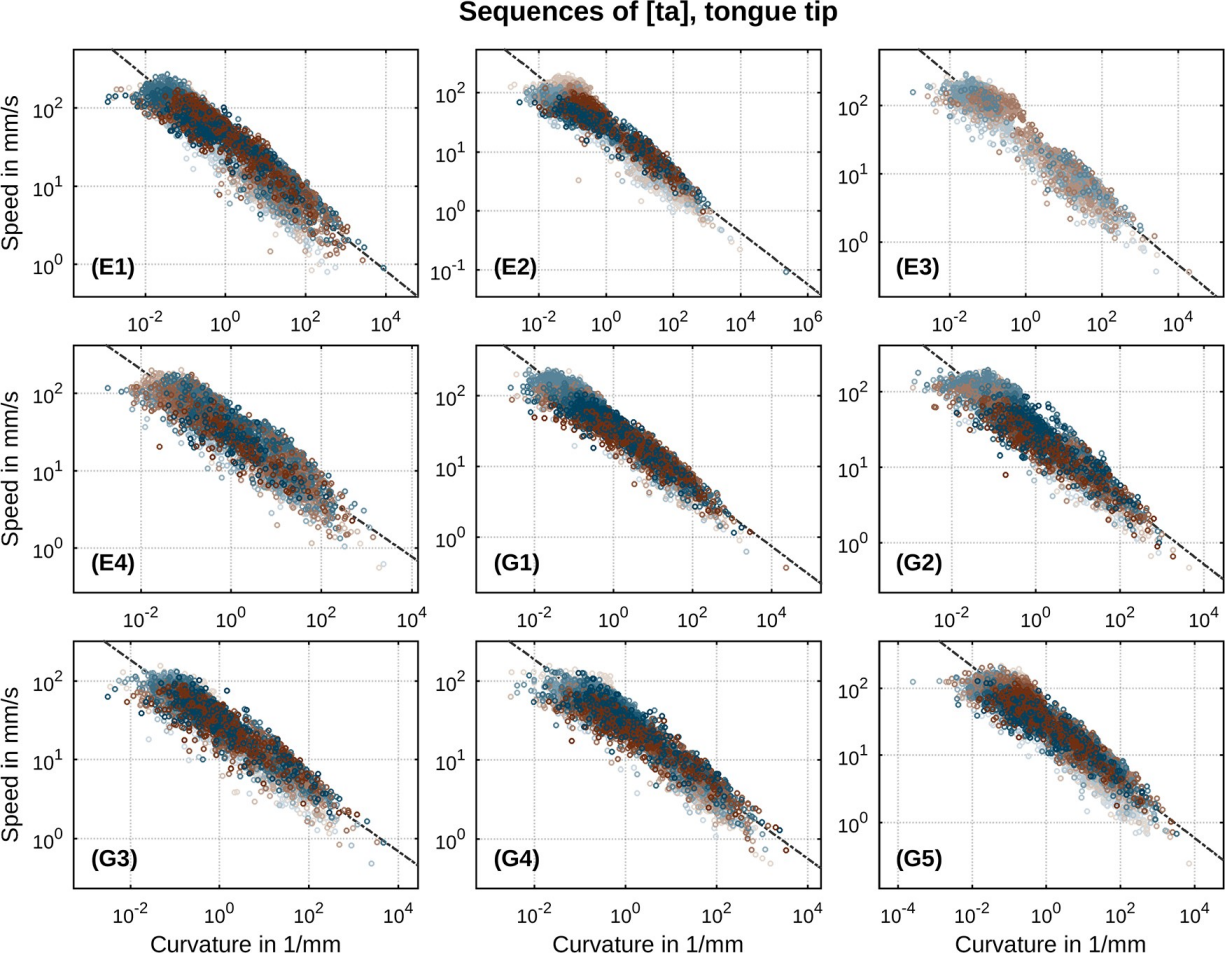
Per-speaker relation between speed and curvature of subsampled movement data in sequences of [ta]. Direction of movement is indicated by color (closing: blue, opening: brown). Metronome rate is indicated by gradual shade (slowest: bright, fastest: dark). Regression lines according to log *v* = log *k* − *β* log *κ* are drawn as black lines.

**Fig 7:**
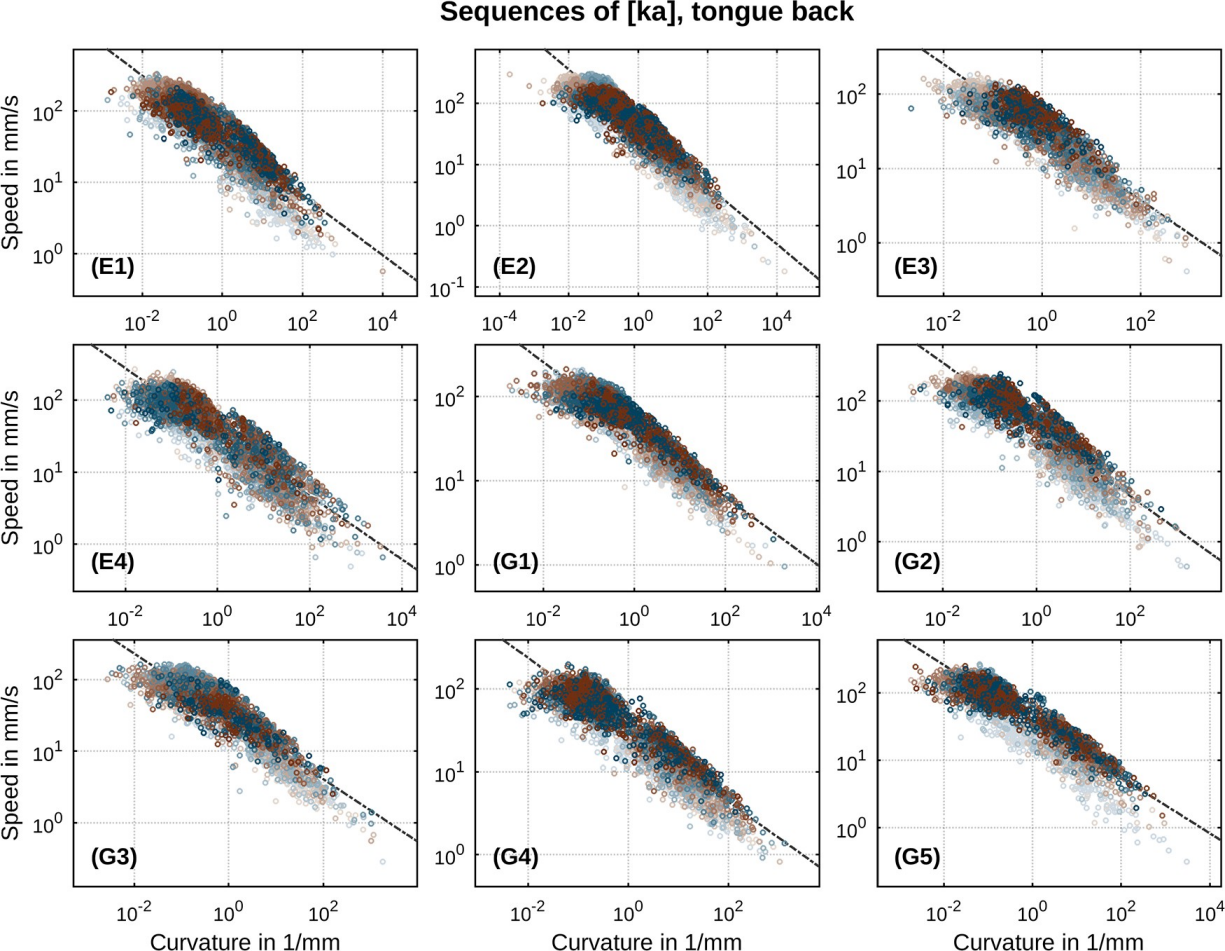
Per-speaker relation between speed and curvature of subsampled movement data in sequences of [ka]. Direction of movement is indicated by color (closing: blue, opening: brown). Metronome rate is indicated by gradual shade (slowest: bright, fastest: dark). Regression lines according to log *v* = log *k* − *β* log *κ* are drawn as black lines.

A first global overview of speed-curvature relations in our data is presented in Figs 6 and 7: each panel is a per-speaker log-log scatter plot of the two essential parameters, speed and curvature. These depictions offer a global overview because, for now, we pool across our entire spectrum of rates within each speaker’s data. In turn, this enables one to see that our experimental paradigm has provided more than ample variation in the two essential parameters whose relation is being assessed here. It is clear, in other words, that we are dealing with a very wide range of speeds and curvatures. It is also clear from this overview that, for each speaker, type of sequence ([ta] or [ka]) and direction of movement (closing or opening), there is evidence for a strong (log-log) correlation between movement speed and trajectory curvature. Individual regression lines according to (6) are drawn in black color. Determined values of the power law’s exponent *β* are in the range of 0.40…0.46 (with correlation strengths *r*^2^ = 0.84…0.93) in case of [ta] and 0.40…0.48 (*r*^2^ = 0.78…0.87) in case of [ka]. Velocity gain factors *k* attain values in the range of 24.92…37.44 for sequences of [ta] and 30.68…45.61 for sequences of [ka]. All *p*-values reside below 0.0001 implying strongest significance. These results indicate the clear presence of a general power law relation between speed and curvature in our data. Recalling that *v* = *kκ*^−*β*^, with instantaneous speed of movement *v* and trajectory curvature *κ*, a *β* of 0.46 implies that every time curvature increases by, for example, a factor of 10, the velocity decreases by a factor of roughly one third or 10^−0.46^ = 0.34. The proportional change in velocity is thus independent of the value of curvature *κ*. This property of size-independence is the hallmark of a power law and implies that the principles organizing and constraining the speech motor system are invariant over variations in kinematic properties and the specifics of the effectors executing the speech movements.

**Fig 8:**
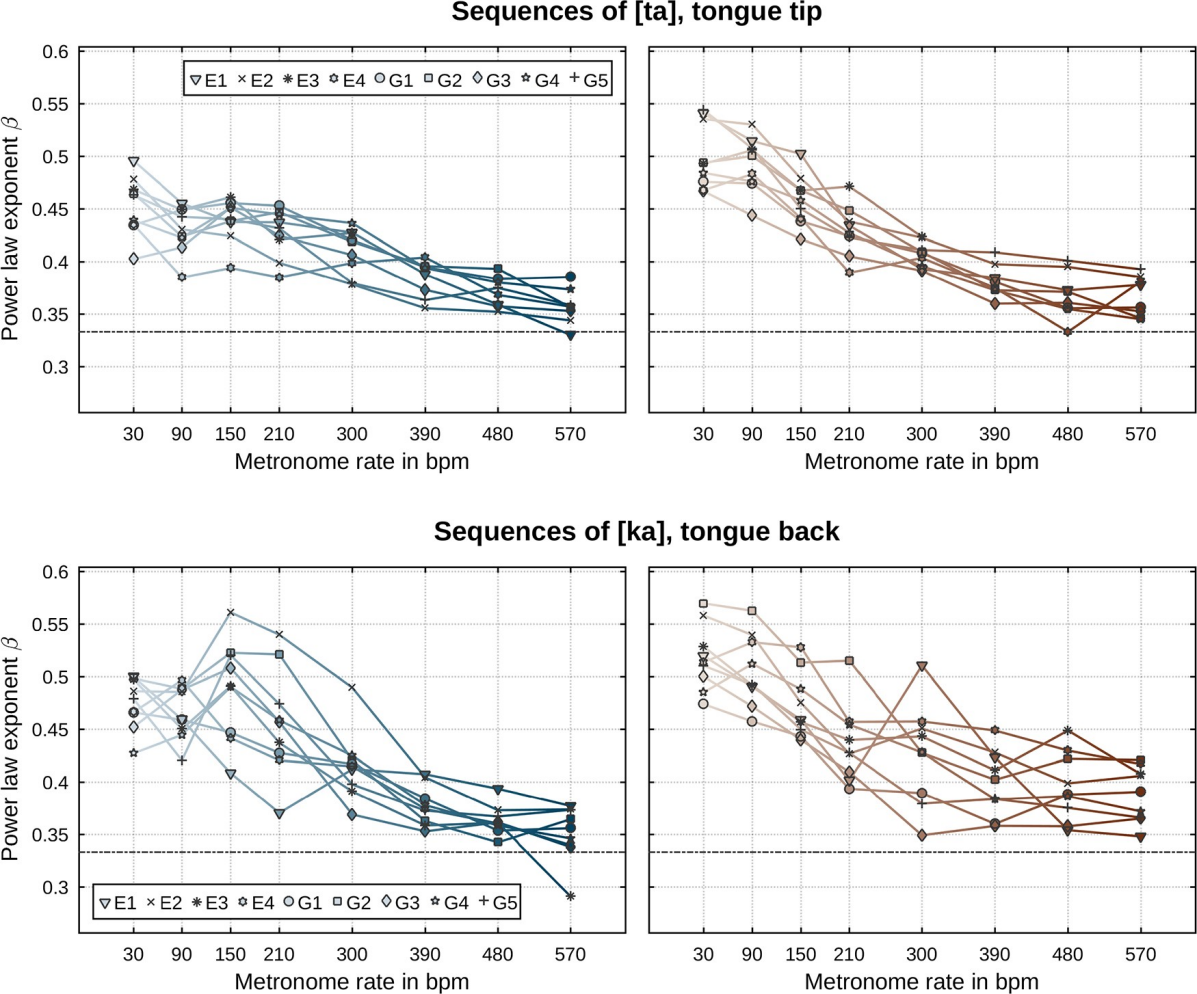
Power law exponents *β* per speaker (symbols) and metronome rate (abscissa). The commonly reported constant of one-third is indicated by a horizontal dashed line. (Top row) Sequences of [ta]. (Bottom row) Sequences of [ka]. (Left column) Closing movements. (Right column) Opening movements.

**Fig 9:**
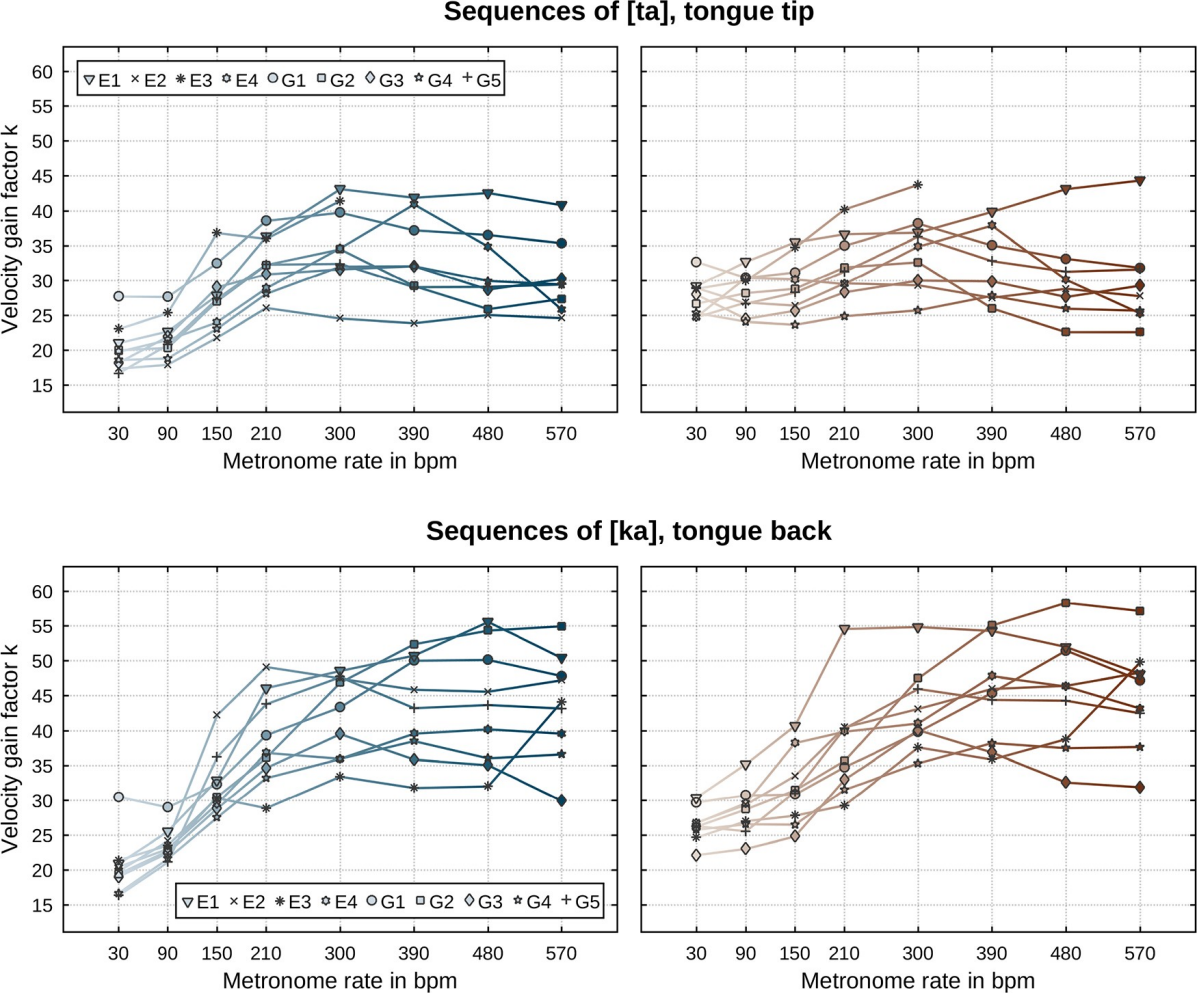
Velocity gain factors *k* per speaker (symbols) and metronome rate (abscissa). (Top row) Sequences of [ta]. (Bottom row) Sequences of [ka]. (Left column) Closing movements. (Right column) Opening movements.

We now proceed to a rate- and direction-specific analysis of our data. The same regression analysis as described above was performed separately for each metronome rate and movement direction. Fig 8 shows the so-determined power law exponents *β*. It is apparent that there is a substantial dependency of the exponent on the induced speech rate. For any type of movement ([ta] and [ka], closing and opening), the per-speaker values of the power law’s exponents at the slowest rate (30 bpm) attain values significantly higher than the commonly reported value of one third but fairly approximate that value as metronome rate increases. There are minor differences with respect to movement direction and identity of the articulator: decreases of *β* values are slightly steeper for opening movements than for closing movements and exponents of [ka] sequences attain slightly larger values than [ta] sequences. Values of the per-speaker determined velocity gain factors, as shown in Fig 9, exhibit a clear rate dependency. That is, values of *k* increase with increasing metronome rate and saturate at faster rates. At faster rates, velocity gain factors for [ka] sequences attain values slightly larger than those for [ta] sequences. There are no notable differences between the two directions of movements in terms of *k*. Table 2 lists all determined correlation strengths *r*^2^ of the power law. For any performed regression, the correlations’ *p*-values reside below 0.0001 indicating strongest significance. The overall range of *r*^2^ values is 0.76…0.97 (median: 0.92) in case of [ta] and 0.54…0.96 (median: 0.89) in case of [ka] sequences. There is a moderate trend for these values to decrease with induced speech rate. Sequences of [ta] attain *r*^2^ values slightly larger than those of [ka].

**Table 2:** Pearson correlation coefficients *r*^2^ of full, three-dimensional data separated by speaker, movement direction and metronome rate. All *p*-values reside below 0.0001 indicating strongest significance.

	E1	E2	E3	E4	G1	G2	G3	G4	G5
[ta] closing, 30 bpm	0.94	0.94	0.94	0.89	0.90	0.95	0.83	0.91	0.92
[ta] closing, 90 bpm	0.92	0.94	0.95	0.87	0.93	0.93	0.89	0.94	0.94
[ta] closing, 150 bpm	0.90	0.95	0.96	0.87	0.95	0.92	0.94	0.95	0.95
[ta] closing, 210 bpm	0.91	0.95	0.96	0.85	0.97	0.94	0.95	0.94	0.94
[ta] closing, 300 bpm	0.93	0.94	0.95	0.86	0.96	0.91	0.93	0.92	0.92
[ta] closing, 390 bpm	0.93	0.92	n/a	0.84	0.94	0.86	0.85	0.90	0.86
[ta] closing, 480 bpm	0.91	0.92	n/a	0.77	0.90	0.82	0.85	0.90	0.88
[ta] closing, 570 bpm	0.79	0.89	n/a	0.76	0.89	0.79	0.83	0.87	0.84
[ta] opening, 30 bpm	0.95	0.96	0.96	0.95	0.97	0.95	0.91	0.94	0.94
[ta] opening, 90 bpm	0.93	0.95	0.94	0.95	0.94	0.95	0.93	0.92	0.94
[ta] opening, 150 bpm	0.91	0.95	0.93	0.90	0.95	0.94	0.94	0.94	0.95
[ta] opening, 210 bpm	0.91	0.94	0.91	0.88	0.95	0.94	0.94	0.94	0.94
[ta] opening, 300 bpm	0.91	0.93	0.91	0.87	0.95	0.92	0.93	0.93	0.92
[ta] opening, 390 bpm	0.91	0.93	n/a	0.84	0.93	0.88	0.85	0.90	0.87
[ta] opening, 480 bpm	0.90	0.92	n/a	0.78	0.86	0.85	0.86	0.90	0.88
[ta] opening, 570 bpm	0.83	0.89	n/a	0.80	0.86	0.84	0.83	0.85	0.87
[ka] closing, 30 bpm	0.92	0.92	0.92	0.88	0.93	0.95	0.93	0.91	0.91
[ka] closing, 90 bpm	0.88	0.94	0.93	0.87	0.89	0.91	0.90	0.92	0.90
[ka] closing, 150 bpm	0.85	0.94	0.88	0.86	0.92	0.90	0.90	0.90	0.95
[ka] closing, 210 bpm	0.76	0.92	0.85	0.85	0.89	0.93	0.87	0.90	0.92
[ka] closing, 300 bpm	0.70	0.86	0.77	0.86	0.90	0.88	0.82	0.90	0.92
[ka] closing, 390 bpm	0.82	0.83	0.72	0.81	0.88	0.81	0.78	0.90	0.91
[ka] closing, 480 bmp	0.92	0.80	0.71	0.79	0.85	0.76	0.66	0.85	0.90
[ka] closing, 570 bpm	0.86	0.74	0.54	0.74	0.83	0.72	0.73	0.84	0.89
[ka] opening, 30 bpm	0.91	0.96	0.94	0.90	0.92	0.94	0.94	0.94	0.91
[ka] opening, 90 bpm	0.88	0.96	0.93	0.88	0.90	0.94	0.92	0.93	0.88
[ka] opening, 150 bpm	0.89	0.94	0.88	0.90	0.92	0.94	0.88	0.93	0.92
[ka] opening, 210 bpm	0.81	0.89	0.85	0.88	0.90	0.94	0.85	0.92	0.92
[ka] opening, 300 bpm	0.77	0.87	0.79	0.85	0.92	0.90	0.80	0.93	0.93
[ka] opening, 390 bpm	0.82	0.87	0.75	0.83	0.92	0.87	0.77	0.92	0.92
[ka] opening, 480 bpm	0.88	0.85	0.77	0.79	0.90	0.82	0.70	0.91	0.92
[ka] opening, 570 bpm	0.84	0.79	0.67	0.76	0.88	0.79	0.76	0.88	0.89

These rate- and direction-specific results both support and sharpen previous results on the speed-curvature power law in a number of ways. Across all metronome rates and the two directions of movement, there exists a highly robust (log-log) correlation between movement speed and trajectory curvature: correlation strengths (see Table 2) attain values significantly higher than those reported in any previous work (Tasko & Westbury: 0.60…0.68, Perrier & Fuchs: 0.49…0.67, Neufeld & van Lieshout: 0.71…0.72, Tomaschek et al.: 0.68…0.73). This result seems, in one part, attributable to our enhanced data representation utilizing three-dimensional signals with advanced numerical differentiation. Regression results from our data intentionally projected to the mid-sagittal plane (2D) still show evidence for the presence of the conjectured power law, but with correlation strengths falling by 5–10 percentage points in comparison to those determined from the full, three-dimensional data; see Table 3 which shows *r*^2^ values of mid-sagittally projected data in comparison to Table 2 of full, three-dimensional data. Relatedly, in some previous works on the speed-curvature power law in speech, it has been reported that the (log-log) correlation between speed and curvature notably weakens for smaller curvature *κ* values. Thus, two previous studies on the speed-curvature relation, Tasko & Westbury [31] and Tomaschek et al. [34], excluded data points with extremal values of curvature from their analyses (Tasko & Westbury: *κ* < 0.006mm^−1^, Tomaschek et al.: *κ* < 10^−4^mm^−1^ and *κ* > 10^3^mm^−1^). Tomaschek et al. [34] even suggested a non-linear relation between (log) speed and (log) curvature. Visual inspection of the speed-curvature relation as shown in Fig 7 may appear to suggest for some speakers (e.g., G5, [ka]) a non-linear relation. However, segregation by rate, as pursued in our analysis, reveals a much more orderly picture. As we have demonstrated, exponents *β* exhibit a clear systematic dependency on metronome rate with strong significance. Different values of the power law’s exponent *β* correspond to different slopes of the linear relation between log speed and log curvature. It is thus a consequence of this fact that the data, when pooled across multiple rates, may appear to show some tendency for nonlinearity. Due to the systematicity of the observed rate-dependency of *β* (small values of curvature correspond to high speeds which in turn are primarily realized at faster rates corresponding to smaller values of *β*), this tendency is an artifact of superposition of different slopes. When a rate-specific analysis is performed, small curvature values do not indicate any deviation from the (log-log) linearity between speed and curvature. Additionally, in computing curvature, numerical issues are amplified precisely at small curvature values. This is so due to instabilities in numerical estimation when $\dot{r}$ and $\ddot{r}$ in (5) are nearly linearly dependent vectors. Two vectors are said to be linearly dependent if they point in the same direction. The cross product of two vectors, which is implicated in computing curvature, vanishes or is very close to zero when the vectors are (nearly) linearly dependent and machine rounding issues become important. Hence, a robust algorithm along the lines described around (5) is called for. In sum, adoption of the natural dimensionality of the data (no reduction to 2D) and advanced numerical differentiation methods indicate no need to either exclude subsets of curvature values or to abandon linearity of the general law relating speed and curvature. As we have seen, in fact, stronger evidence than that reported by any previous work on the law is found when all data is included.

**Table 3:** Pearson correlation coefficients *r*^2^ of intentionally mid-sagittally projected data (2D) separated by speaker, movement direction and metronome rate. All *p*-values reside below 0.0001.

	E1	E2	E3	E4	G1	G2	G3	G4	G5
[ta] closing, 30 bpm	0.87	0.89	0.87	0.84	0.85	0.92	0.79	0.90	0.86
[ta] closing, 90 bpm	0.85	0.90	0.90	0.80	0.87	0.87	0.86	0.91	0.91
[ta] closing, 150 bpm	0.83	0.89	0.93	0.82	0.90	0.88	0.87	0.90	0.91
[ta] closing, 210 bpm	0.86	0.88	0.92	0.78	0.93	0.91	0.90	0.89	0.89
[ta] closing, 300 bpm	0.88	0.88	0.89	0.80	0.92	0.84	0.88	0.86	0.87
[ta] closing, 390 bpm	0.89	0.85	n/a	0.77	0.90	0.81	0.79	0.84	0.81
[ta] closing, 480 bpm	0.87	0.86	n/a	0.70	0.86	0.72	0.81	0.84	0.80
[ta] closing, 570 bpm	0.67	0.82	n/a	0.67	0.81	0.71	0.78	0.82	0.76
[ta] opening, 30 bpm	0.91	0.92	0.93	0.84	0.92	0.94	0.85	0.89	0.91
[ta] opening, 90 bpm	0.88	0.93	0.87	0.89	0.89	0.91	0.91	0.88	0.91
[ta] opening, 150 bpm	0.84	0.90	0.87	0.85	0.92	0.89	0.89	0.85	0.91
[ta] opening, 210 bpm	0.87	0.88	0.86	0.83	0.90	0.92	0.89	0.88	0.89
[ta] opening, 300 bpm	0.88	0.88	0.89	0.80	0.92	0.87	0.88	0.86	0.87
[ta] opening, 390 bpm	0.87	0.87	n/a	0.76	0.88	0.81	0.76	0.83	0.82
[ta] opening, 480 bpm	0.85	0.87	n/a	0.70	0.81	0.78	0.82	0.81	0.82
[ta] opening, 570 bpm	0.73	0.82	n/a	0.67	0.77	0.80	0.79	0.80	0.79
[ka] closing, 30 bpm	0.88	0.88	0.90	0.82	0.85	0.90	0.85	0.81	0.85
[ka] closing, 90 bpm	0.83	0.89	0.86	0.78	0.82	0.87	0.82	0.85	0.82
[ka] closing, 150 bpm	0.80	0.94	0.80	0.83	0.86	0.84	0.82	0.85	0.92
[ka] closing, 210 bpm	0.70	0.88	0.77	0.83	0.81	0.88	0.80	0.85	0.88
[ka] closing, 300 bpm	0.59	0.80	0.69	0.80	0.81	0.83	0.74	0.85	0.87
[ka] closing, 390 bpm	0.72	0.78	0.64	0.77	0.80	0.76	0.67	0.84	0.86
[ka] closing, 480 bmp	0.86	0.73	0.64	0.69	0.79	0.66	0.57	0.77	0.85
[ka] closing, 570 bpm	0.79	0.65	0.47	0.61	0.75	0.64	0.57	0.77	0.82
[ka] opening, 30 bpm	0.87	0.91	0.90	0.84	0.86	0.91	0.89	0.91	0.86
[ka] opening, 90 bpm	0.80	0.89	0.85	0.80	0.80	0.85	0.81	0.90	0.81
[ka] opening, 150 bpm	0.84	0.90	0.76	0.85	0.86	0.88	0.80	0.90	0.82
[ka] opening, 210 bpm	0.77	0.82	0.76	0.85	0.81	0.89	0.79	0.88	0.84
[ka] opening, 300 bpm	0.69	0.82	0.71	0.81	0.84	0.88	0.71	0.87	0.88
[ka] opening, 390 bpm	0.73	0.83	0.67	0.78	0.85	0.84	0.70	0.85	0.86
[ka] opening, 480 bpm	0.84	0.77	0.73	0.66	0.84	0.73	0.55	0.84	0.87
[ka] opening, 570 bpm	0.77	0.66	0.62	0.67	0.85	0.75	0.57	0.80	0.79

Previous work on the power law in speech sometimes reports differences in the values of exponents *β* and velocity gain factors *k* with respect to the identity of the articulator (e.g., [31]). However, these differences do not seem to hold robustly across studies. Thus, Perrier & Fuchs [32] report that the direction of variation of *β* across effectors was speaker-dependent and that the significance of differences in *k* substantially reduces when segmented movements are considered (ibidem, p. 23). In our data, we find some evidence for articulator-related differences in *β* only at the slower rates and for *k* only at the faster rates (see Figs 8 and 9). Specifically, at rates below approx. 300–390 bpm we observe that exponents of the tongue tip articulator (in sequences of [ta]) attain slightly smaller values than those of the tongue back articulator (in sequences of [ka]). For metronome rates above or equal to approx. 300–390 bpm, we find somewhat higher velocity gain factors for the tongue tip than the tongue back. One reason for the lack of agreement here may be that the results of Tasko & Westbury [31] are based on a “broad form of analysis” (ibidem, p. 74) where movements of sensors placed on the orofacial structures were evaluated regardless of whether the effector (on which the sensor was attached to) was involved actively in the forming of a speech gesture. All speech gestures involve movement, but not all movements are part of a gesture. For example, the lower lip moves upward during the formation of the closure required for the first consonant in [ta], but that movement is a passive consequence of the jaw which rises (in unperturbed conditions) to aid in effecting the tongue tip constriction. In our work, we evaluate the law for effector movements in which that effector is actively engaged in the production of a speech gesture, e.g., the tongue body movement during the forming of the constriction and its release in [ka]. This is in keeping with the literature from other areas of motor control where, for instance, in evaluating the law for planar drawing tasks, it is the effector actively implicated in the act of drawing whose conformity to the law is evaluated. Our main result on the validity of the power law along with certain specifics, to be taken up in the next section, show very good agreement with other studies from other areas of motor control. Finally, in congruence with Perrier & Fuchs [32] and Tomaschek et al. [34], we demonstrate that values of the velocity gain factors *k* generally increase with increasing induced speech rate (see Fig 9). This finding is also in agreement with many reports from the field of general motor control [13, 50–52].

## Discussion: From figural constraints to movement dynamics

The foregoing results and specifically the validity of the power law relation between speech and curvature under the rather wide range of rate conditions elicited in our paradigm implies the existence of general principles that organize and constrain the functioning of the speech motor system. In the present section, we turn to consider these results in the context of other work in movement science, considering both commonalities across domains as well as prospects for furthering our understanding of speech.

In the field of general motor control, the speed-curvature law is most often associated with the commonly reported exponent *β* of one third. A clear result from our data is that, in speech, an exponent of one third is approximated only at the faster rates. Slower rates exhibit significantly higher *β* values. As we discuss next, this result finds precursors in the literature on motor control. In turn, this indicates that, over and beyond the existence of the relation between speed and curvature in both speech and other domains, there exist further commonalities, in human movement, across effector systems and the particular modes of their operation. As we also take up next, various features of the performed actions and their lawful modulation as a function of task parameters (specifically, rate in our experiments) may offer potential entries into furthering our understanding of the control systems governing speech movements.

We begin with a brief summary of reports from the non-speech domain pointing to different sources of variability in the power law’s parameters. In a three-dimensional drawing task, Schaal & Sternad [49] reported increasing values of *β* for increasingly sized patterns. Significantly deviating from one third *β* values have also been reported for complex-shaped movements not limited to the class of ellipsoids from which initial evidence for the power law originally derived [22, 53]. Specifically, Huh & Sejnowski [53] have demonstrated that in planar drawing tasks complexity of movement shape is positively related to the value of the exponent *β*, i.e., more complex shapes correspond to higher values of *β*. A different source for the variability in the power law’s exponent has been suggested in terms of constraints under which movements are performed. In a planar drawing task, Wann et al. [51] presented evidence for significantly smaller deviations of the exponent from one third when participants were forced to perform their movements at a faster rate compared to their self-chosen pace. Lastly, Pollick & Ishimura [48] have demonstrated that three-dimensional target-to-target movements are accompanied by significantly larger than one third exponents and conjectured that these deviations from one third may be related to the control regime underlying the generation of movements. Specifically, that work raises the question whether the regime controlling movements for which only the endpoint target locations are specified is the same as in more constrained movements where effectors must follow specific paths.

**Fig 10:**
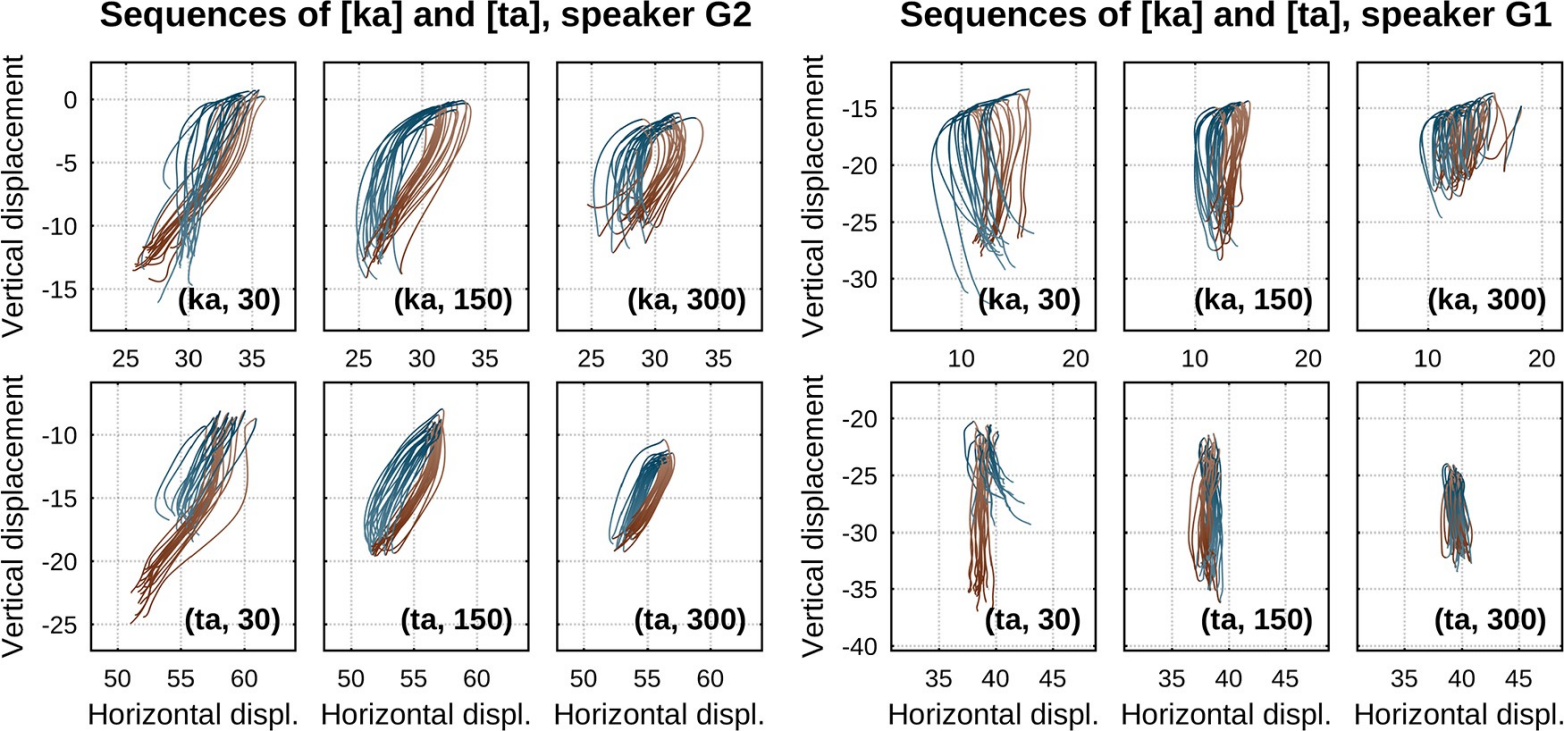
Movement traces of tongue back and tongue tip sensors in sequences of [ka] and [ta] from two speakers at different metronome rates. (Left-hand side) Speaker G2. (Right-hand side) Speaker G1. (Top row) Sequences of [ka]. (Bottom row) Sequences of [ta]. Direction of movement is indicated by color (closing: blue, opening: brown).

Let us place our results in the context of these studies from the non-speech domain. As some of these studies refer to the shapes of trajectories, we present in Fig 10 (left-hand side) traces of tongue back and tongue tip movement patterns by one of our speakers for sequences of [ka] (top row) and [ta] (bottom row) at different metronome rates. By visual inspection of these traces, it is evident that there is a clear decrease in movement excursion extents with increasing metronome rate (from approx. 15 to 10 mm for [ka] and [ta]). In agreement with Schaal & Sternad [49], this reduction in (what [49] refers to as) pattern size is accompanied by a decrease in the power law’s *β* values as demonstrated in our main results section. Furthermore, for both sequences of [ka] and [ta] (Fig 10, left-hand side, top and bottom row), movement patterns exhibit a transition from complex (non-elliptical) shaped traces to simpler elliptical shapes at the faster rates. This observation of a reduction in shape complexity accompanied by decreasing *β* values agrees with reports by Huh & Sejnowski [53]. However, in our data not all speakers exhibited a clear transition from non-simple to simple shapes (but all speakers did exhibit the decrease in *β* values). To wit, movement traces of [ka] and [ta] from another speaker are given in the right-hand side of Fig 10 (top and bottom rows). For this speaker, a transition from complex to simpler shapes is hardly visible (for both [ka] and [ta]). However, excursion extents for both articulators systematically decrease from approx. 15 mm to 10 mm at the faster rates as with the other speaker. In sum, [ta] figural shapes are not identical to [ka] shapes (Fig 10, top vs. bottom rows) and they are individual-specific (Fig 10, left-hand vs. right-hand side). However, there is one rigorous generalization that clearly stands out across [ka] and [ta] and across all individuals: as demonstrated in our main results, there is a clear inverse correlation between metronome rate and the law exponent *β* (Fig 8). *β* decreases with rate. In other words, whereas shape generalization is not so evident across [ka] and [ta] and across speakers, generalization is seen in the systematicity of how the parameterization of the law changes as a function of rate across articulators and speakers.

Before elaborating on this relation between metronome rate and the law exponent *β*, let us consider reasons why finding shape generalizations in our data does not appear as straightforward as in Huh & Sejnowski [53]. First, whereas participants in Huh & Sejnowski [53] were asked to trace planar movements with a single effector, two different effectors are involved in our paradigm, tongue tip and tongue back. Movements of the tongue body implicate deformation of a larger mass than that of the tongue tip. There are strong hints from other areas of biological action that body mass is crucially involved in how movements are organized and effected. For example, in studies of how speed affects locomotion across animals of different sizes, there is evidence that different modes of locomotion occur at similar speeds only when speed is scaled to body mass [54]. Second, unlike in drawing movements of the hand studied in Huh & Sejnowski [53], the tongue moves in the oral cavity. Tongue tip movements for [t] are confined in a space different from that of tongue body movements for [k]. Thus, the presence of a more or less pronounced alveolar ridge and its more posterior palate morphology contribute both to the extent and the direction (horizontal, vertical, lateral) of movements [55]. It is conceivable that taking into account the geometry of the hard structures and in particular palate morphology and the ways in which tongue movements adapt to speaker-specific aspects of that morphology may be crucial in better understanding trajectory shape. For example, Neufeld & van Lieshout [33] consider algorithms for transforming tongue sensor positions into a palate-relative coordinate space, by means of shortest paths to the surface of a speaker’s palate. In the resulting methods, the way in which the trajectory for the tongue tip is transformed is different from that of the tongue back (because the palate has a different shape in the regions where tongue tip versus tongue body actions take place). Effectively, this implies that the geometric shape of tongue tip movements is dependent on the coordinate system chosen. It is thus plausible that using appropriate transformations of the different articulator trajectories may enable identification of shape transitions comparable to those we present in Fig 10 (left-hand side) also for speakers where Cartesian coordinates do not offer evidence for such transitions (right-hand side). Regardless, we emphasize again that even though shape generalizations are not so evident in our data as they may be in other domains [53], there is a robust systematicity of how the law exponent *β* changes as a function of rate (Fig 8). This systematicity holds across articulators and speakers.

Let us, finally, consider our results in the context of Wann et al. [51] and the conjecture in Pollick & Ishimura [48] regarding potential distinctions in the control regime or constraints governing the elicited movements. As demonstrated in our main results, there is a clear inverse correlation between metronome rate and the law exponent *β* (Fig 8) in agreement with the results of [51]: *β* decreases with rate. In the context of Pollick & Ishimura [48] who hint at the potential role of differences in the control regime underlying movement execution, it seems relatively uncontroversial that in our study movements at the slower rates can be considered as target-to-target movements with [k] (or [t]) and [a] as the oppositional consonant-vowel targets. In agreement with [48], we find that these movements (at slower rates) show significantly larger exponents than one third. Just as [48] have raised the question about the presence of a distinction between target-to-target and non-target-to-target movements in terms of the underlying nature of control and corresponding *β* values, we too are faced with the question whether movements at the faster rates with significantly smaller exponents belong to the same class as movements at the slower rates with higher exponents. This potential distinction between qualitative control regimes underlying what may be apparently similar movements has been pursued in work that has so far remained unrelated to the speed-curvature law. Specifically, there is evidence from other areas of motor control indicating that increasing rate may result in qualitative changes in the control regime underlying movements. In a unimanual finger flexion-extension task, Jirsa & Kelso [56] and Huys et al. [57] demonstrated that a dynamical system with distinct control regimes can model the transition from discrete target-to-target movements to rhythmic (cyclic) movements with movement rate as the bifurcation parameter. At the slow rates and specifically below a critical value of rate, finger movements are driven by a control regime governed by fixed point dynamics (with either one or two stable fixed points, depending on whether the motor system instantiates both a flexion-extremum target and an extension-extremum target or just one of these extrema as a target along with a transient excursion away from it). At the faster rates, above the critical value of rate, the control regime switches to a different dynamical organization constituting a limit cycle attractor. It seems plausible that a similar distinction between discrete target-to-target and cyclic (non-targeted) movements separated by a critical speech rate may apply to our data as well.

**Fig 11:**
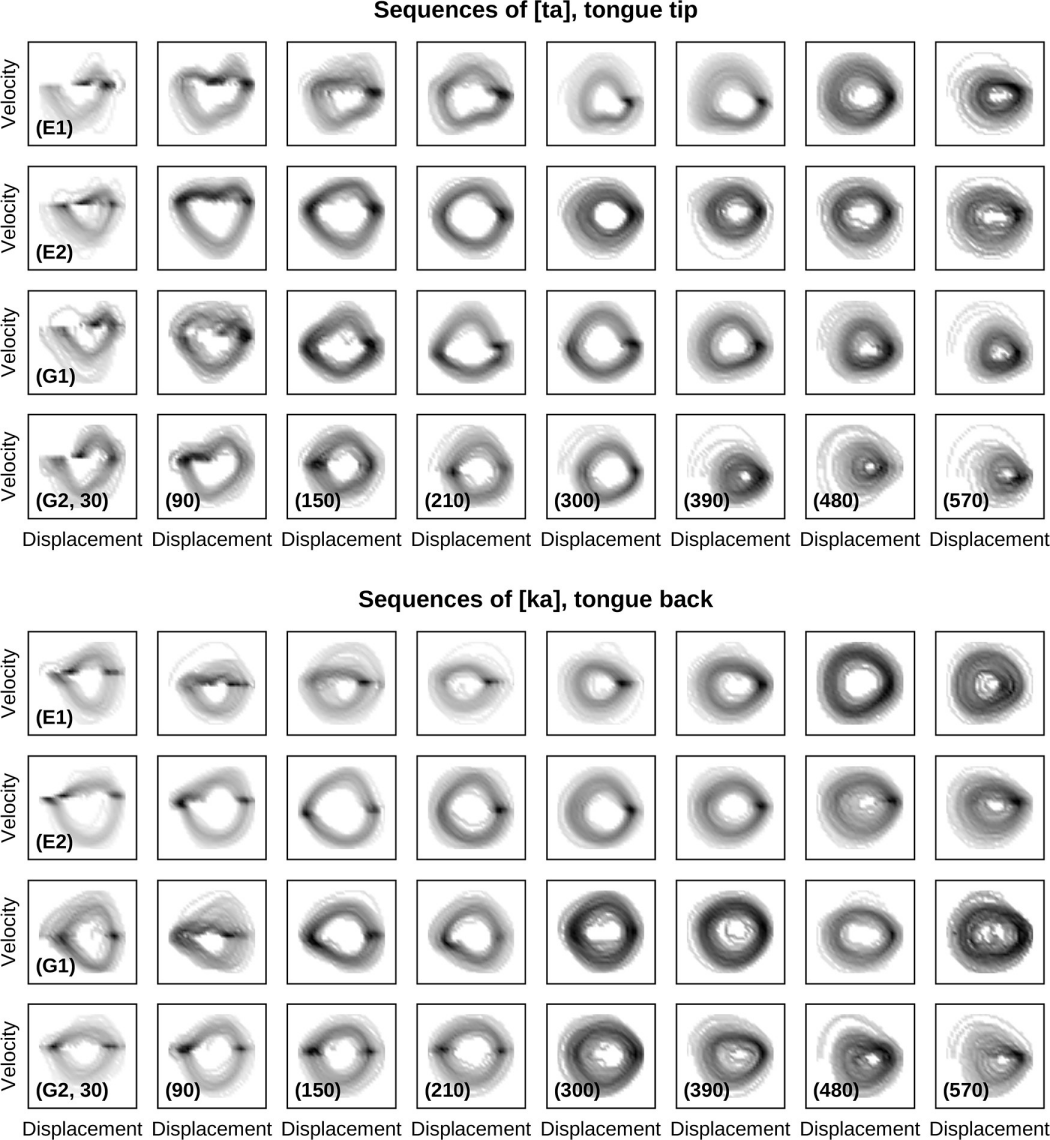
Phase portraits of tongue tip (top panel) and tongue back movements (bottom panel) from four speakers (E1, E2 of English and G1, G2 of German) at different metronome rates (columns). Darker shades indicate more visited states in the phase space. A localized region of frequent visits (dark point-like structures) hints at the presence of a fixed point.

To assess the potential presence of evidence for distinct dynamical regimes governing movements in our data, we turn to consider the phase portraits of these movements as a function of speech rate. For a second-order dynamical system, as in the class of systems entertained in Jirsa & Kelso [56] and Huys et al. [57], phase portraits consist of trajectories in the *x*-$\dot{x}$-plane (displacement and velocity, the two dimensions of the system’s phase space). Fig 11 shows examples of phase portraits of the tongue tip and tongue back articulator’s principal component. These figures are spatio-temporal approximations of the phase space’s density in the vicinity of an effector’s trajectory. Darker shades (higher densities) correspond to more frequently visited phase space states, whereas brighter shades (lower densities) indicate rarely and shortly visited states of the phase space. The attractors of a dynamical system (such as fixed points or limit cycles which classify the governing regime) are structures in phase space towards which every trajectory ultimately evolve to, regardless of its starting point (the so-called initial conditions). It is plausible to assume that the phase space density (number of visiting trajectories) is much higher close to an attractor than far away from it. Thus, the topology of a region of high density can be exploited to identify the topology of the underlying dynamical regime. A localized region of high density (a dark point-like structure) suggests the presence of a fixed point. In contrast, dark ring-like structures suggest the presence of a periodic attractor (e.g., a limit cycle). By inspection of Fig 11, we see that evidence for localized structures indicating the presence of a fixed point can be found only at the slower rates (e.g., [ta] and [ka], speaker G2, 30–210/300 bpm). At faster rates, these structures disappear and dark ring-like structures of high density emerge indicating the presence of (non-fixed point) periodic attractors (e.g., [ta], speaker E2, 300–570 bpm). Furthermore, the same qualitative picture as shown in Fig 11 holds across all our speakers. Hence, there are promising indications for the existence of two distinct control regimes governing speech movements as a function of metronome rate. We plan to purse this evidence further in future work.

Overall, then, the suggestion is that the control regimes for speech may not be limited to target-to-target concatenation. In other words, fixed point dynamics as proposed by the standard model of speech gestures in Eq (3) may not be the only model available to the motor system in effecting speech action. Pursuing this suggestion would require rigorous assessment of candidate dynamical systems that can encompass more than a single control regime. Models with this property exist (e.g., [56, 58]) but have not been explored in speech yet.

## Conclusion

The present article examined the speed-curvature power law in speech, using an experimental paradigm explicitly designed to elicit a wide range of speeds, from extremely slow to extremely fast. In this paradigm, we tracked the trajectories of sensors attached to the tongue tip and tongue back, the major lingual effectors involved in the production of the syllables [ta] and [ka] respectively. Analysis of our data in their full three-dimensions and using advanced numerical differentiation methods offers stronger evidence, as judged by correlation strengths, for the speed-curvature power law than that reported in previous studies devoted to its assessment. Furthermore, we demonstrate the presence of a clear speech rate dependency of the power law’s instantiation in terms of its parameter values. Specifically, the often-sought or reported exponent of one third in the statement of the law is unique to a subclass of movements which in the case of speech correspond to just a small range of rates under which a particular utterance is produced. Comparison of our results to findings from other areas of motor control indicates that there is potential for lawful relations between rate and the geometric shape of speech movements to be revealed. It remains to be seen whether a thorough analysis of the movements’ geometric properties in the domain of speech supports that indication and what lessons such analysis may offer for speech and non-speech biological action. Finally, our results and specifically the rate dependencies of the power law’s exponent values hint at the possibility of a multiplicity or at least non-uniqueness in the control regimes controlling speech movements. We plan to pursue both of these indications in future work.
